# Enteric Coated Pellets with Lactoferrin for Oral Delivery: Improved Shelf Life of the Product

**DOI:** 10.3390/pharmaceutics17010023

**Published:** 2024-12-26

**Authors:** Nika Kržišnik, Blaž Grilc, Robert Roškar

**Affiliations:** Faculty of Pharmacy, University of Ljubljana, Aškerčeva Cesta 7, 1000 Ljubljana, Slovenia; nika.krzisnik@ffa.uni-lj.si (N.K.);

**Keywords:** lactoferrin, pellets, enteric coating, gastric digestion, stability, shelf life

## Abstract

Background/Objectives: Lactoferrin (Lf), a multifunctional iron-binding protein, has considerable potential for use as an active ingredient in food supplements due to its numerous positive effects on health. As Lf is prone to degradation, we aimed to develop a formulation that would ensure sufficient stability of Lf in the gastrointestinal tract and during product storage. Methods: A simple, efficient, and well-established technology that has potential for industrial production was used for the double-coating of neutral pellet cores with an Lf layer and a protective enteric coating. Results: The encapsulation efficiency was 85%, which is among the highest compared to other reported Lf formulations. The results of the dissolution tests performed indicated effective protection of Lf from gastric digestion. A comprehensive stability study showed that the stability was similar regardless of the neutral pellet core used, while a significant influence of temperature, moisture, product composition, and packaging on the stability of Lf were observed, and were therefore considered in the development of the final product. The experimentally determined shelf life is extended from 15 to almost 30 months if the product is stored in a refrigerator instead of at room temperature, which ensures the commercial applicability of the product. Conclusion: We successfully transferred a technology commonly used for small molecules to a protein-containing product, effectively protected it from the destructive effects of gastric juice, and achieved an acceptable shelf life.

## 1. Introduction

Lactoferrin (Lf) is an 80 kDa, single-chain, iron-binding, basic, globular glycoprotein of the transferrin family with ~700 amino acids [1,2]. It is present in the milk of many mammals and is also found in various other mucosal secretions and in the secondary granules of neutrophils [3,4]. Due to its specific structural properties, it reversibly binds Fe^3+^ ions with high affinity and over a wide pH range [5]. In addition to iron binding, Lf has various biological functions, including antimicrobial, anti-inflammatory, immunomodulatory, antitumor, antioxidant, wound-healing and bone-regenerating effects [1,2]. Due to its beneficial effects on human health, Lf is commercially extracted from milk or whey and added as a functional ingredient to food, pharmaceutical and cosmetic products [3,6,7]. It can be used for iron supplementation, immune system enhancement, supporting the gut microbiota and limiting bacterial growth (e.g., for traveler’s diarrhea), maintaining oral hygiene (e.g., caries, periodontitis), and treating skin and mucosal lesions [8,9,10]. In addition, it has no toxic adverse effects and was approved as a Generally Recognized as Safe substance and as a novel food ingredient [7,11].

Two major challenges hinder the widespread supplementation of Lf. First, Lf is prone to degradation and is sensitive to various processing factors that significantly affect product quality. The main factors affecting the stability of Lf are temperature, pH and ionic strength of the medium, iron saturation, high pressure, and the presence of polysaccharides or proteins. Iron-saturated form is more resistant to heat and proteolysis [11]. Differences in glycosylation patterns or salt concentration affect the aggregation behavior of Lf [12]. Secondly, Lf is very susceptible to an acidic environment and proteolytic enzymes in the stomach, with a half-life of less than 0.5 h [1,4,13]. While the gastrointestinal system is immature in infants, allowing Lf to withstand gastric digestion, Lf is rapidly digested in adults, preventing its interaction with Lf receptors in the small intestine [4]. Many of the functional properties of Lf are highly dependent on its structural integrity. Therefore, the protection of Lf from the destructive effects of gastric juice and its delivery to the site of action is a crucial issue [4,14]. Several approaches have been proposed to improve oral bioavailability and stability, increase permeability, and achieve targeted delivery of Lf [5,15,16]. Commonly used approaches include iron saturation, nano- or microencapsulation, PEGylation, and the addition of absorption enhancers [15,16,17]. These approaches have several drawbacks, such as insufficient protection of Lf from gastric digestion, questionable safety, high cost, complex manufacturing, low loading efficiency, and/or instability [4,16,17,18,19,20]. Encapsulation efficiency, which is crucial for cost-effectiveness, is usually below 80%, often even below 60% [4,15,18,19,21,22,23,24,25]. Moreover, many formulations have issues with insufficient protection of Lf in gastric juice, as 15–80% of Lf is released or degraded in dissolution tests, which is above the pharmacopoeial limit [7,20,21,25]. However, not only the stability of Lf formulations under gastric conditions, but also the *in vitro* stability is a very important aspect, as the shelf life of the product is crucial for its commercial applicability. A literature review revealed a lack of data on the storage stability of Lf products, as most researchers do not focus on this critical quality attribute. Recent studies demonstrated that nanostructured lipid carriers, chitosan-based nanoparticles and liposomes containing Lf were stable at 4–37 °C for 2–3 months. However, in these studies, only the physical stability of the formulation (size, particle size distribution, zeta potential) was considered, while the Lf content was not evaluated [23,24,26,27]. Another study showed that Lf is more stable when encapsulated in solid lipid particles than in liposomes. Nevertheless, less than 30% of the intact Lf remained in both formulations after 6 months of storage at 25 °C [18].

The aim of our work was to develop an innovative Lf formulation that effectively protects Lf from gastric digestion using a well-established and common technology used in the formulation of classic medicines containing small-molecule active ingredients. Particular attention was paid to ensuring the stability of the product and determining its shelf life, as this is crucial for the cost-effectiveness of the product. In addition, high process efficiency, scale-up capability, and commercial applicability of the process were also considered.

## 2. Materials and Methods

### 2.1. Materials

#### 2.1.1. Materials Used for the Preparation of Pellets with Lactoferrin

Neutral pellet cores Cellets 200 were purchased from HARKE Pharma GmbH (Mülheim an der Ruhr, Germany) and PharSQ^®^ Spheres CM were kindly donated by Budenheim KG (Budenheim, Germany). Lf was supplied by Arhel d.o.o. (Komenda, Slovenia). Lf was isolated from whey using ion-exchange chromatography. The chromatographic purity of Lf was 82.9%, the absolute purity determined using the Lf standard was 82.1%, and the iron saturation expressed as the A value was 4.3%, while the C value was 53.2%. Pharmacoat 606 was purchased from Shin-Etsu (Tokyo, Japan). Polyethylene glycol 6000, and triethyl citrate were purchased from Sigma-Aldrich (St. Louis, MO, USA). Tween^®^ 80 was purchased from Merck (Darmstadt, Germany). Eudragit^®^ L30-D55 was purchased from Evonik Industries (Essen, Germany). Glycerol monostearate was purchased from ACEF Spa (Fiorenzuola d’Arda, Italy). Purified water was produced at the University of Ljubljana, Faculty of Pharmacy (Ljubljana, Slovenia). Size 0 hard capsules were purchased from Farmalabor Srl (Canosa di Puglia, Italy), and alu-alu packaging foil was purchased from Logapak d.o.o. (Slovenska Bistrica, Slovenia).

#### 2.1.2. Materials Used for the Characterization of Pellets with Lactoferrin

Microcrystalline cellulose (MCC) powder Avicel PH 200 was purchased from HARKE Pharma GmbH (Mülheim an der Ruhr, Germany). Potassium dihydrogen phosphate, trisodium phosphate dodecahydrate, concentrated hydrochloric acid, solid sodium hydroxide, sodium hydroxide for 1 M solution (Titrisol^®^), trifluoroacetic acid, and Millex^®^-GV PVDF filters (0.22 μm) were purchased from Merck (Darmstadt, Germany). Standard of lactoferrin from bovine colostrum (≥85%) was purchased from Sigma-Aldrich (St. Louis, MO, USA). High purity Milli-Q^®^ water was obtained using a Milli-Q^®^ A10 Advantage water purification system (Millipore Corporation, Bedford, MA, USA). HPLC-grade acetonitrile, anhydrous methanol (Hydranal™—Methanol Dry), and titration solution for coulometric Karl Fischer titration (Hydranal™—Coulomat AG) were purchased from Honeywell (Charlotte, CA, USA).

### 2.2. Preparation of Lactoferrin Pellets

The Lf formulation was prepared in a two-stage pellet coating process using the bottom-spray fluid bed coater GPCG1 (Glatt GmbH, Binzen, Germany). The fluid bed system was equipped with a peristaltic pump (Flocon 1B.1003-R/65, Petro Gas Ausrüstungen Berlin GmbH, Berlin, Germany), an air compressor (Compak 3/90, Omega Air d.o.o., Ljubljana, Slovenia) and a dehumidifier (Inštitut Zoran Rant, Škofja Loka, Slovenia).

First, the pellet cores were coated with an Lf layer, and in the second step, an enteric coating (EC) was applied. MCC cores and calcium phosphate (CaP) cores were coated to obtain two different products. The inlet air was conditioned to 30% relative humidity (RH). A 1.2 mm coaxial spray nozzle was used. The delimiting cylinder was located 15 mm above the distribution plate. The Lf coating dispersion contained 17.5% (*w*/*w*) Lf, 2.1% (*w*/*w*) Pharmacoat 606, 0.4% (*w*/*w*) polyethylene glycol 6000, and 80% (*w*/*w*) purified water. Pharmacoat 606, polyethylene glycol 6000 were dissolved in purified water. The solution was mixed using a propeller stirrer Eurostar 20 (IKA-Werke, Staufen im Breisgau, Germany) equipped with R 1342 four-bladed propeller stirrer at 600 rpm. Once completely dissolved, solid Lf was added and mixed until completely dissolved.

The second layer consisted of the EC based on the polymer Eudragit^®^ L. The dispersion was prepared according to the official procedure recommended by the manufacturer. The dispersion contained 55.2% (*w*/*w*) Eudragit^®^ L30-D55, 1.7% (*w*/*w*) triethyl citrate, 0.8% (*w*/*w*) glycerol monostearate, 1.0% (*w*/*w*) Tween^®^ 80, and 41.3% (*w*/*w*) purified water. The process parameters for both coatings are listed in Table 1. The efficiency of the coating process was determined by calculating the dry matter yield of the pellets using Equation (1). After coating, the pellets were sieved through a 1100 µm sieve to remove the agglomerated pellets. The finished pellets were filled into size 0 hard capsules and sealed in alu-alu packaging. The uniformity of dosage units (mass variation) was tested according to European Pharmacopoeia.

(1) $$Coating\;yield\;[\%]=\frac{m_{p}\times \left(\frac{100\;-\;{RH}_{p}}{100}\right)-m_{c}\times \left(\frac{100\;-\;{RH}_{c}}{100}\right)}{m_{s}}\times 100$$

*m_p_*—mass of coated pellets; *m_c_*—mass of pellet cores; *m_s_*—mass of solids applied; *RH_p_*—percentage of moisture in coated pellets; *RH_c_*—percentage of moisture in pellet cores.

### 2.3. Particle Size Distribution

The particle size distribution of the pellets was measured by laser diffraction (Mastersizer 3000, Malvern Panalytical Ltd., Malvern, UK) using the Aero S air dispersing unit. The measurement was carried out at an air pressure of 1.2 bar for dispersing the pellets and a feed rate of 25% of the dosing ramp. The valid measurement obscuration range was set to 0.5–6.0%. The average measurement of three repetitions was given as the result. The size distribution of the pellets was determined at each production step to obtain information about the thickness of the coating.

### 2.4. Raman Mapping of Pellet Cross-Section

A sample of Lf pellets with an MCC core was mixed with MCC powder. The mixture was compressed using a tableting press (SP300, IMA Kilian GmbH & Co. KG, Köln, Germany). Flat punches with a diameter of 12 mm were used to produce the tablets. A relatively low compression force of 1.5 kN was used to avoid damaging the pellets. The prepared tablets were cut precisely into slices of 30 µm at −20 °C using a cryostat Leica CM1850 (Leica Biosystems Nussloch GmbH, Deer Park, IL, USA). Once the pellet core was visually exposed, the tablet was transferred to a Raman microscope. The cross-section was imaged in 6478 points with a step size of 3 µm. Raman spectra in the range of 111–1804 cm^−1^ were recorded at 20× magnification using a 785 nm laser with an acquisition time of 10 s in three repetitions for each point. The spectra were centered at 1500 cm^−1^ using the 600 grating. The obtained spectra were preprocessed with a rubber band baseline correction, a Savitzky Golay filter at window seven, a polynomial order of three, and normalized with vector normalization.

### 2.5. Dissolution of Enteric Coated Pellets

Dissolution studies were performed in Vankel VK7000 (Agilent Technologies, Santa Clara, CA, USA) USP II (paddle) apparatus according to the European Pharmacopoeia procedure for delayed-release solid dosage forms. The temperature was maintained at 37 ± 0.5 °C throughout the experiment, and the paddle rotation speed was set at 100 rpm (MCC pellets) or 150 rpm (CaP pellets). The dissolution studies were performed in two phases. For the simulation of gastric fluids, 750 mL of 0.1 M hydrochloric acid solution (pH 1.0) was used as the dissolution medium. After 120 min, the passage from the stomach to the small intestine was simulated by adding 250 mL of 0.2 M trisodium phosphate dodecahydrate solution to raise the pH to 6.8. If necessary, the pH was adjusted with sodium hydroxide solution or hydrochloric acid solution to obtain the desired pH. Samples were collected at predetermined time points (5, 30, 60, 120, 126, 156, 186, 246, and 306 min) using a 2 mL syringe. Samples were filtered through (Millex^®^-GV, 0.22 μm), with the first milliliter of the sample removed as waste. The withdrawn sample volume was replaced by the appropriate medium after each sampling. The concentration of dissolved Lf was determined by a reversed-phase HPLC method (see Section 2.7). The average dissolution profile was calculated and presented with the standard deviation.

### 2.6. Stability Study

Various samples (Table 2) were included in the stability study. The samples were stored in a refrigerator (4 °C) and in climate chambers under accelerated (40 °C, 75% RH) and long-term (25 °C, 60% RH) storage conditions. Dissolved samples from time zero were stored at controlled room temperature (up to 25 °C). Samples were analyzed at predetermined time points (0, 12, 26, 45, and 52 weeks for long-term conditions and refrigerator; 0, 2, 4, 8, 12, 26, 45, and 52 weeks for accelerated conditions; and 0, 2, 4, 8, 12, and 26 weeks for dissolved samples) according to ICH Q5C guidelines slightly modified by the inclusion of additional time points.

Samples were prepared by accurately weighing 30–32 mg of spray-dried Lf, 70–73 mg of MCC or CaP pellets without EC, and 83–86 mg of EC MCC or CaP pellets, and dissolving them in 25 mL of 50 mM phosphate buffer (pH 6.8) in a volumetric flask. One liter of phosphate buffer was prepared from 6.8 g of potassium dihydrogen phosphate, 22 mL of 1 M sodium hydroxide solution, and Milli Q^®^ water up to 1 L. The pellets were mixed on a magnetic stirrer for 2 h. All samples were filtered (Millex^®^ GV, 0.22 µm) prior to analysis. Each sample was prepared in duplicate, except 5 parallels that were analyzed at time zero.

### 2.7. Sample Analysis

An Agilent 1100/1200 series HPLC system (Agilent Technologies, Santa Clara, CA, USA) with diode array detector and ChemStation data acquisition program was used for sample analysis (dissolution, stability, content). The validated stability-indicating reversed-phase HPLC method developed by Osel et al. was used [28]. Briefly, the analysis was performed on a BioZen™ Intact XB-C8 column (150 × 4.6 mm, 3.6 μm; Phenomenex, Torrance, CA, USA) at 30 °C with gradient elution using 0.1% (*v*/*v*) trifluoroacetic acid in Milli-Q^®^ water and 0.1% (*v*/*v*) trifluoroacetic acid in acetonitrile as the mobile phase at a flow rate of 1.0 mL/min. The selected detection wavelength was 280 nm.

### 2.8. Water Content

The moisture content of the pellets was determined by the thermogravimetric method. The loss on drying of the pellets was measured at 85 °C for 60 min using a HR83-P moisture balance (Mettler Toledo, Greifensee, Switzerland). The pellets (3–5 g) were evenly distributed on the measuring pan. The percentage decrease in sample mass after drying was reported as the result. The moisture content was used for the calculation of the coating yield (Equation (1)).

Karl Fischer coulometric titration was used to determine the water content in our formulations during the stability study at time points 0, and 26 weeks. Four milliliters of anhydrous methanol were injected into the sealed test tubes containing 25–30 mg of the sample. The test tubes were shaken for 5 min (Vibromix 10, Tehtnica, Železniki, Slovenia), and then sonicated for 15 min (Sonis 4, Iskra PIO, Šentjernej, Slovenia). Each sample was prepared in duplicate. Aliquots of 0.5 mL were injected into the titration vessel (SI Analytics^®^ TitroLine^®^ 7500 KF trace, Module 1, Xylem Analytics Germany Sales GmbH & Co. KG., Weilheim, Germany) filled with titration solution. Measurements were performed in triplicate for each test tube. A blank sample was prepared in the same way as the samples and measured twice. The result of the blank sample was subtracted from the sample result. The water content in the sample (Equation (2)) was expressed as a percentage of the sample weight (% *w*/*w*).

(2) $$Water\;content\;[\%\;w/w]\;=\;\frac{W_{s}\;\times \;m_{s+m}\;-\;W_{m}\;\times \;m_{m}}{10,000\;\times \;m_{s}}$$

*W_s_*—measured water content in the sample [μg/g]; *W_m_*—measured water content in the blank sample [μg/g]; *m_s+m_*—sum of the masses of the sample with Lf and the anhydrous methanol [mg]; *m_m_*—mass of anhydrous methanol added to the sample with Lf [mg]; *m_s_*—mass of the sample with Lf [mg].

### 2.9. Data Analysis

GraphPad Prism 10 (Dotmatics, Boston, MA, USA) was used to create the graphs. Zero-, first-, and second-order kinetics were fitted to Lf degradation using least-squares regression (MS Excel 2019). Determination coefficients were used to evaluate models and the model with the highest determination coefficient was selected. Subsequently, the rate constants were calculated from the most adequate model. If less than 30% of the initial Lf content remained in the sample, the corresponding time points were not considered when calculating the constants. Confidence intervals (95%) for the rate constants were calculated using MS Excel 2019 (Analysis TookPak). The shelf life was calculated as the time point at which the lower 95% confidence interval for the Lf content intersects the specification limit, which was set at 80% of the initial Lf content. The lower confidence interval was calculated according to Bolton and Bon [29].

## 3. Results

### 3.1. Coating Process

The Lf formulation was produced in a two-stage pellet coating process. The first layer applied to the neutral pellet core (MCC or CaP) was an Lf layer. The second layer was an EC based on the polymer Eudragit^®^ L. The process parameters, such as air flow, nozzle pressure, dispersion feed rate, and temperatures, were optimized for each coating during formulation development. The coating yield was calculated to be 85% for MCC pellets and 86% for CaP pellets. The thickness of the Lf coating was determined by subtracting the average diameters of the EC pellets and pellets without EC. The results are shown in Table 3. It was found that the thickness of the Lf coating was almost twice as high for CaP pellets as for MCC pellets, even though the same mass of coating was applied. This is due to the size and density of the pellet core. Since the CaP pellets are about 1.5 times larger and have a higher density than MCC pellets, the total particle count was lower compared to MCC pellets. This limits the surface area available for coating, which resulted in a thicker coating when CaP cores were used. Since the coating yields and the masses of coating dispersion sprayed were comparable, it can be assumed that the coating thickness is inversely proportional to the available surface area.

**Figure 1:**
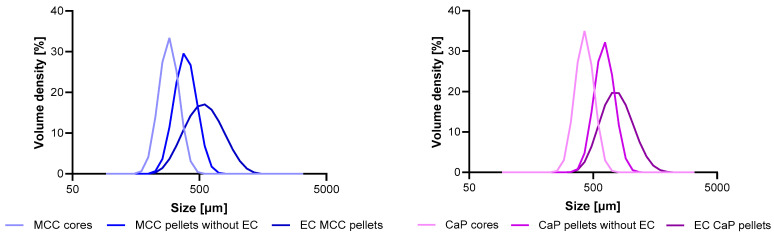
Particle size distribution after each coating applied for MCC (**left**) and CaP (**right**) cores.

The width of the particle size distribution remained similar after the first coating, and only the average diameter of the pellets increased (Figure 1). The EC (Table 3) were of the same thickness for both pellet types, as the coating was adjusted to the diameter of the pellets in order to obtain the same function of the protective layer. However, after the application of the EC, the particle size distribution widened (Figure 1). Ideally, the distribution should not widen after coating, as this could indicate poor uniformity of the coating. Nevertheless, the dissolution results (see Section 3.3) show that the coating is consistent with the delayed-release dosage forms.

The percentage of agglomerates determined by sieving the product through a 1100 μm sieve. The amounts of agglomerates were low, namely 0.1% for the Lf coating and 0.4% for the EC. To obtain the final product, the EC pellets were filled into gelatin capsules to facilitate administration and to ensure appropriate dosage of the pellets, and the capsules were heat sealed into alu-alu packaging to protect Lf from environmental factors. The uniformity of dosage units test showed that the capsules met the pharmacopoeial requirements for mass variation, as the acceptance value was 4.7 and 9.5 for MCC and CaP pellets, respectively, which is below the pharmacopoeial limit (L1) of 15. Furthermore, no unit exceeded the maximum acceptable range for deviation (L2).

### 3.2. Raman Mapping of MCC Pellet

**Figure 2:**
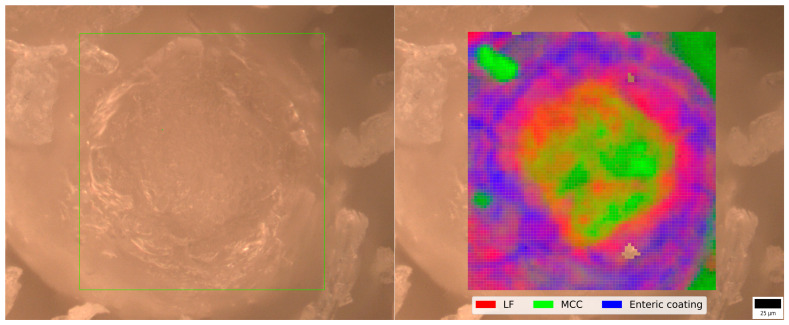
Raw image (**left**) and overlaid image (**right**) of a pellet cross-section showing the distribution of Lf and EC.

Raman mapping of the pellet cross-section revealed the expected structure, which was obtained by two-stage coating the pellets. The overlay of the pellet core is shown in Figure 2. The green color stands for the MCC, the red color for the Lf layer, and the blue color for the EC. The color map shows the relative intensity of the characteristic peaks in the Raman spectrum that belong to the individual components. The characteristic peak for MCC was at 1097 cm^−1^, for Lf at 1006 cm^−1^, and for EC at 766 cm^−1^.

### 3.3. Dissolution of Enteric Coated Pellets

**Figure 3:**
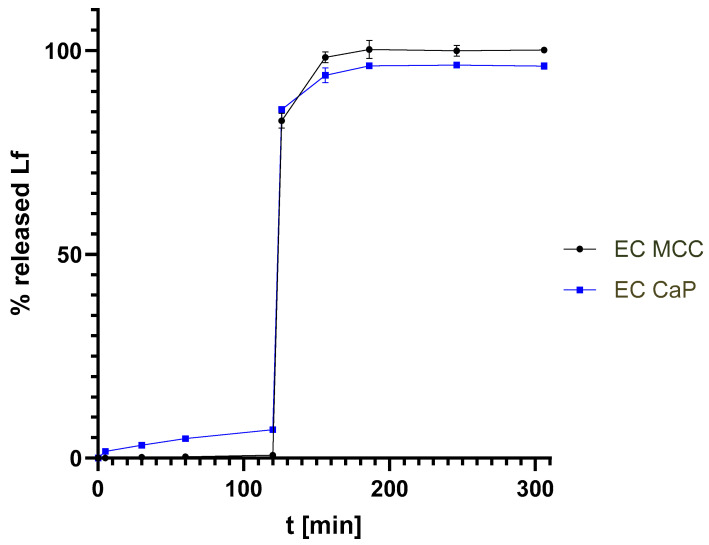
Dissolution profiles (European Pharmacopoeia, method A) of Lf pellets with EC.

The dissolution of the prepared pellets complies with the European Pharmacopeia requirements for delayed-release dosage forms. During the acid stage, less than 10% of the Lf was released from both pellet types (Figure 3), namely 0.7% for EC MCC pellets and 7.0% for EC CaP, with no individual value exceeding 10%. After increasing the pH, the release of Lf was immediate, reaching 82.8% and 85.6% of the total Lf dose in only 6 min after the media change for EC MCC and EC CaP pellets, respectively. At the end of the dissolution test, 100.1% and 96.2% of Lf was released for EC MCC and EC CaP pellets, respectively. That complies with the specification that at least 80% of the total amount of active substance must be released in both the acid and buffer stages. The results of the parallel experiments were comparable. The RSD values were below 15% in the acid stage and below 3% in the buffer stage. Pellets with CaP cores performed slightly worse in the acid stage. This was due to technical problems, as the higher density of the pellets caused them to settle to the bottom of the vessel and stick together. To ensure that the concentration of Lf in the samples was high enough for quantification (0.05–5.0 mg/mL), a mass of pellets about 20 times larger than that contained in a capsule was used for the release test. For this reason, and also because of gastric motility, we assume that the difference between the two types of pellets would be irrelevant in vivo.

### 3.4. Storage Stability

Different samples (Table 2) were included in the stability study under three different storage conditions to evaluate the effects of the pellet core, EC, gelatin capsule, primary packaging, temperature, and RH on Lf stability. In addition to the final products, raw materials (spray-dried Lf) and intermediate products (pellets without EC) were included as controls. Some samples were without packaging, while others were filled into gelatin capsules, and some were additionally protected by alu-alu packaging. Several kinetic models were fitted to the Lf degradation. The determination coefficients were highest for the zero-order kinetic model, which is consistent with our previous study [28]. Therefore, it was used to determine the rate constants, which are listed in Table 4.

The influence of the product composition on the stability of Lf was investigated. For this purpose, two types of neutral pellet cores, namely MCC and CaP, were used. The results of the stability study show that there is no significant difference in the stability of Lf with one or the other type of pellet core, as the 95% confidence intervals for the rate constants overlap in most cases (Table 4). The influence of EC was evaluated by comparing the stability of Lf in pellets with or without EC under accelerated storage conditions (40 °C, 75% RH). The rate constants for pellets with EC were almost twice as low as for pellets without EC when no additional protection was used (Table 4). Thus, the EC protects Lf to a certain extent (stability improved by a factor of approximately 2), however, the opposite effect was observed when the pellets were additionally protected by a capsule or primary packaging, indicating a general destabilizing effect of the EC. This destabilizing effect is further confirmed by the results of Lf stability in the dissolved samples, as the rate constants for pellets with EC are significantly higher than for uncoated pellets (Table 4). On the other hand, there is no significant difference between the samples with and without capsules when they were protected by the alu-alu packaging (Table 4). The capsules are primarily intended for adequate dosing of the pellets, although the capsule itself has a stabilizing effect if the samples are not additionally protected by the packaging.

**Figure 4:**
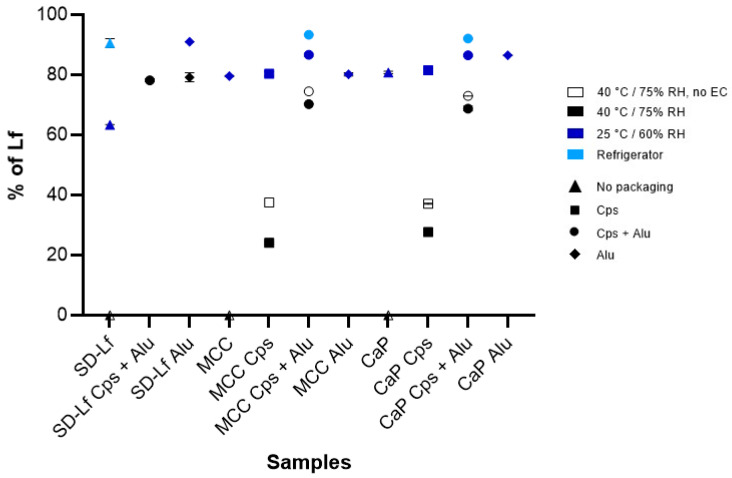
The percentage (±SD) of Lf remaining in the samples after 1 year of storage under different storage conditions and in different packaging.

Our results confirm that environmental factors, such as temperature and moisture, have a significant impact on the stability of Lf. In general, the rate constants were highest for accelerated conditions (40 °C, 75% RH) and lowest for samples stored in a refrigerator (4 °C) (Table 4). The differences were most pronounced for the samples that were not protected by the packaging, as no Lf was present in these samples after 1 year of storage under accelerated conditions (Figure 4). The spray-dried Lf sample was the most sensitive to the accelerated conditions, as no Lf remained in the sample after only two months. Furthermore, solid-state aggregation of Lf occurred and Lf no longer dissolved in the phosphate buffer. The rate constants for the unprotected samples were more than an order of magnitude higher under accelerated conditions (40 °C, 75% RH) than under long-term storage conditions (25 °C, 60% RH) (Table 4). When the pellets were filled in a capsule or protected by primary packaging, this effect was less pronounced. The stability of Lf improved by a factor of 1.5 when the product was stored in a refrigerator instead of at room temperature (Table 4). Since temperature has an important impact on the stability of Lf, it was also considered when optimizing the coating process. The product temperature during the process should be between 35 and 40 °C to ensure adequate mechanical properties and sufficient drying of the coatings and at the same time not affect the stability of Lf.

Another environmental factor that significantly affects the stability of Lf in the product is moisture. For the samples that were not packed in alu-alu packaging, which are impermeable to gasses and moisture, the moisture content in the samples increased significantly (Table 5), and the rate constants were higher, indicating that Lf was less stable when it was not protected from moisture. This is further confirmed by the sample of pellets with EC in an alu-alu packaging that was unexpectedly opened during the stability study, as the stability of Lf in this sample was similar to the sample without the alu-alu packaging (Table 4). Therefore, a specification limit of maximum allowed percentage moisture content in the final product was set at 5%. The results of the stability study show a significant stabilizing effect not only of the primary packaging but also of the capsules, which is particularly evident under accelerated conditions (40 °C, 75% RH). Interestingly, this effect is greater for pellets without EC, where the rate constant was reduced by a factor of more than 8 simply by filling the pellets into gelatin capsules (Table 4). The reason for this is probably that the moisture penetrates the Lf layer more slowly due to the capsule. The effect of water on the stability of Lf is also demonstrated by dissolved samples. Lf in the dissolved samples of pellets with EC stored at controlled room temperature was five times less stable than Lf in the same pellets stored in a climate chamber at 25 °C. Interestingly, the stability of the dissolved spray-dried Lf sample was similar to the stability of the spray-dried Lf powder at the same storage temperature.

**Figure 5:**
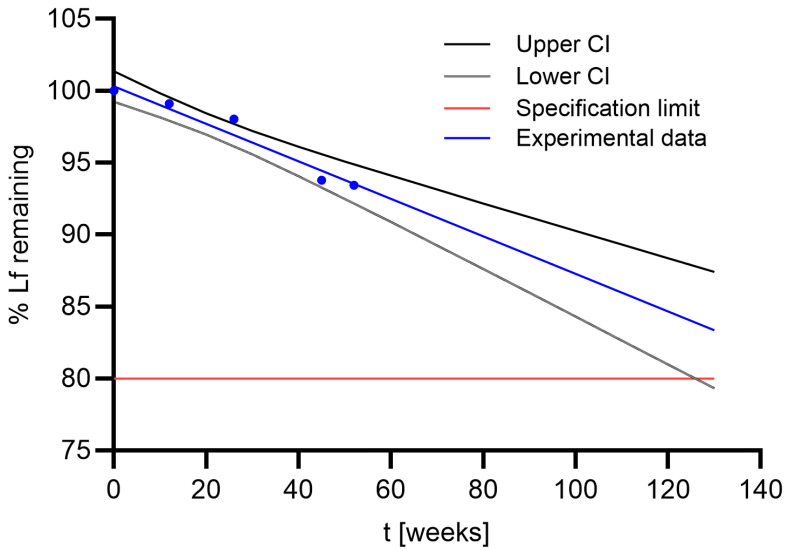
Experimental determination of shelf life using 95% confidence interval for EC MCC pellets containing Lf stored in refrigerator (4 °C).

The shelf life of the final product (pellets with EC filled in capsules and packed in alu-alu packaging) was determined on the basis of the stability study results (Table 6). The time at which the lower 95% confidence interval intersects the specification limit of 80% of the initial Lf content in the product was calculated (Figure 5). The shelf life under long-term storage conditions (25 °C, 60% RH) was 1.3 years and 1.6 years for pellets with MCC and CaP cores, respectively. When stored in the refrigerator, the shelf life for both the product with MCC core and the product with CaP core was 2.4 years. The shelf life can therefore be extended by approximately one year if the product is stored in the refrigerator instead of at room temperature.

## 4. Discussion

Lf is a multifunctional protein with many positive effects on health, which make it interesting for use as a food supplement. However, as a protein, Lf is susceptible to degradation due to processing factors [11] or the acidic environment and proteolytic enzymes in the stomach [1,4]. Therefore, it is crucial to develop a formulation that effectively protects Lf from gastric digestion and enables safe delivery of Lf to the site of action. Accordingly, our aim was to develop such a formulation using a well-established technology. In addition, our focus was also on ensuring the storage stability and determining the shelf life of the product as this is crucial for the commercial applicability of the product.

The innovative Lf formulation was prepared by multistage film coating of neutral pellet cores in a Wurster chamber, which is widely used in the pharmaceutical industry for the preparation of small molecule dosage forms for oral administration. However, it is generally not used for protein drugs, as proteins are usually administered parenterally and are therefore formulated in the form of liquid dosage forms or lyophilizates [30]. However, Lf requires local delivery to the small intestine, and therefore, solid oral dosage forms can be used. Accordingly, we recognized the potential of the former technology for the development of a formulation with Lf that safely delivers Lf to the small intestine. The first layer applied to the core was an Lf layer made with an aqueous solution of Lf and a binder. The second layer was an EC applied over the Lf layer. The widely used polymer Eudragit^®^ L was chosen for the EC, as it ensures gastroresistance due to its pH-dependent solubility. Such coatings are commonly used for conventional drugs to protect the drug from the acidic pH and enzymatic activity in the stomach [31]. The prepared pellets were filled into gelatin capsules to facilitate administration and to ensure appropriate dosage of the pellets. One capsule contains 200 mg of Lf. The capsules were heat sealed into alu-alu packaging to protect Lf from environmental factors, such as light, oxygen, and moisture. The experiments to increase the batch size were also carried out. Only minor changes to the process parameters were required to move from the laboratory stage to the pilot stage of pellet production. In one coating process, 1.3 kg of pellets with Lf were produced, which is sufficient for about 2500 capsules. The results of the scale-up experiment show that the production of our product on an industrial scale is feasible and that there is potential for commercialization of the product.

During formulation development, the process parameters were optimized for each coating. The major concern was the stability of Lf under the selected conditions of the pellet coating process. Collisions of pellets and elevated temperatures during coating could damage Lf. Therefore, the main focus during the coating process was on maintaining an appropriate temperature of the product, which should be between 35 and 40 °C. Such temperatures are not problematic for Lf due to the short duration of the manufacturing process. Nevertheless, the formation of degradation products of Lf was monitored during the formulation development using the stability-indicating reversed-phase HPLC method [28]. We were able to confirm that no or only negligible amounts of Lf degradation products were formed during the coating process. Furthermore, only 0.5% of the produced pellets were discarded due to agglomeration. The losses of Lf during the coating process were rather low compared to other reported Lf formulations. The encapsulation efficiencies were 85% and 86% for MCC and CaP pellets, respectively. Encapsulation efficiencies of more than 80% are rarely reported in the literature [27], and are often even below 60% [4,15,18,19,21,22,23,24], especially nanoparticulate carrier systems, which are associated with low loading efficiency and particle aggregation due to thermodynamic instability [16]. Process efficiency is particularly important when expensive active ingredients are used, which is the case for Lf compared to other common active ingredients in food supplements, such as vitamins and minerals.

To evaluate the quality of the product, a comprehensive characterization of the final product was carried out. Our results confirm that the size of the pellets (MCC vs. CaP) does not affect the quality of the product. Raman spectroscopy confirmed that the desired structure of the product with two distinct layers was obtained. The EC covers the Lf layer and thus protects it. This is further confirmed by the dissolution results, as less than 10% of the Lf was released in the acid stage, which meets the requirements of the European Pharmacopoeia, in contrast to many reported modern delivery systems (e.g., liposomes, microparticles, microcapsules) [4,7,20,21,25]. The release profiles were adequate also for the pellets produced in the scale-up experiment. This means that, in our product, Lf is effectively protected from the acidic pH and enzymatic degradation in the stomach. The protection of Lf in the stomach is of utmost importance as acid hydrolysis and pepsin cleavage can occur [13]. Moreover, protonation at acidic pH can lead to a loss of bound iron, resulting in a change in conformation, making Lf more susceptible to proteolysis [32]. Due to the EC, our formulation enables the safe delivery of Lf into the small intestine. There the higher pH increases the solubility of Eudragit^®^ L and almost all Lf is released, allowing it to interact with the Lf receptors in the small intestine and exert its beneficial effects. The protection of Lf in gastric juice was adequate in some formulations of the beads prepared by Cao et al. However, only 20–60% of the dose was released in simulated intestinal fluid within five hours, which also means that the formulation does not meet the pharmacopoeial requirements for delayed-release dosage forms [19].

When developing a product, not only the stability under in vivo conditions is important, but also the storage stability, as this is essential for the economic viability of the product. In the literature, this aspect is insufficiently considered for products with Lf [4,7,18,19,20,21,22,23,24,25,33]. For this reason, our aim was to investigate the factors that influence stability and, based on the results, to create the final product and determine its shelf life. The influence of the pellet core (MCC or CaP), the EC, and the protection (capsules and/or primary packaging) on the stability of Lf was investigated. The product was stored under three different conditions to test the effects of temperature and RH on the stability of Lf. In most cases, there were no significant differences in stability between the different pellet cores. Interesting was the contradictory effect of the EC, which protects Lf to a certain extent when no additional protection is used. However, when the pellets are stabilized by capsules or primary packaging, a destabilizing effect of EC is observed. On the contrary, capsules have no effect when the product is properly stabilized (alu-alu packaging), and have a stabilizing effect on Lf in pellets if they are not packed in primary packaging. Therefore, the main role of capsules is to facilitate the administration and dosing of pellets. Obtained results show that environmental factors have a significant impact on the stability of Lf, therefore, the use of appropriate packaging is essential for improving the stability of Lf. Moisture has a negative effect on the stability of Lf and can also lead to agglomeration of the pellets, which has a negative effect on the EC and can lead to an inadequate release profile. Therefore, the specification limit for the maximum moisture content in the final product was set at 5% to ensure the stability of Lf and adequate performance of the product. For the above reasons, the final product requires a suitable packaging material, such as alu-alu packaging, which is impermeable to moisture, and gasses. Another important factor is temperature, which affects both the choice of process parameters and the storage stability. Elevated temperature has a significant effect on the stability of Lf, e.g., it was found that storing the product in the refrigerator significantly extends the shelf life of the product.

The shelf life was determined experimentally for the final product, i.e., the EC pellets filled in capsules and packed in impermeable packaging, with Lf being the most stable. In general, the regulatory requirements for food supplements are less stringent than for medicines. The shelf-life specification was set to 80% of the initial Lf content, as the guidelines of EU Directive 2002/46/EC for vitamins allow content deviations in an interval between 80% and 150% of the declared content. The calculation of the shelf life was based on a 95% confidence interval, which is generally used in the pharmaceutical industry to determine the shelf life of medicines. The value is therefore stricter and more conservative than if the shelf life is calculated without the confidence interval (Table 6). The stability of our product was experimentally confirmed and the shelf life was calculated based on the results of the stability study. This is a very important aspect, as the shelf life is crucial for the commercial applicability of the product. However, most researchers do not evaluate storage stability as they only focus on the stability of Lf formulations under gastric conditions. In the few cases, where storage stability was considered, Lf was significantly less stable than in our product. Lf in liposomes was completely degraded after 3 to 5 months, and in the case of solid lipid particles more than 70% of Lf was degraded after 6 months at room temperature [18]. Therefore, such formulations are not commercially applicable. On the other hand, the shelf life of our product is at least 15 months at room temperature. When the product is stored in the refrigerator, the stability is even better and a shelf life of almost two and a half years was achieved, which is remarkable for a food supplement containing a protein.

## 5. Conclusions

Enteric-coated Lf pellets were prepared by two-stage coating of neutral pellet cores in a Wurster chamber. A simple, innovative and most importantly effective approach was chosen to protect Lf from unfavorable conditions in the stomach. The pellets with Eudragit^®^ L coating proved to be gastroresistant, enabling targeted delivery of Lf to the small intestine. In addition, the high yield of the manufacturing process is an advantage over other delivery systems. The proposed manufacturing technology can be applied not only for Lf but also for many other active food ingredients that need to be protected from enzymes and acidic conditions in the stomach or local administration in the small intestine. A comprehensive characterization of the product confirmed its quality, which was also maintained during large-scale production. This is one of the first studies to address the stability of Lf during product development and storage. Moreover, an experimentally determined shelf life of more than two years was achieved under appropriate storage conditions, making the product commercially viable.

## Figures and Tables

**Table 1:** Process parameters for two-stage coating of neutral pellet cores with a lactoferrin (Lf) layer and enteric coating (EC).

Coating Type	Inlet Temperature [°C]	Product Temperature [°C]	Outlet Temperature [°C]	Air Flow [m^3^/h]	Nozzle Pressure [bar]	Dispersion Feed Rate [g/min]
Lf layer	60	35	35	75	1.8	16
EC	47	29	29	75	1.8	12

**Table 2:** The storage conditions and Lf samples included in the stability study.

Samples		Storage Conditions			
		40 °C 75% RH	25 °C 60% RH	4 °C	Dissolved up to 25 °C
**Spray-dried Lf**	No packaging	×	×	×	×
	Cps + Alu	×			
	Alu	×	×		
**MCC pellets without EC**	No packaging	×			×
	Cps	×			
	Cps + Alu	×			
**CaP pellets without EC**	No packaging	×			×
	Cps	×			
	Cps + Alu	×			
**EC MCC pellets**	No packaging	×	×		×
	Cps	×	×		
	Cps + Alu	×	×	×	
	Alu		×		
**EC CaP pellets**	No packaging	×	×		×
	Cps	×	×		
	Cps + Alu	×	×	×	
	Alu		×		

Abbreviations: microcrystalline cellulose (MCC), calcium phosphate (CaP), enteric coating (EC), relative humidity (RH), gelatin capsules (Cps), alu-alu packaging (Alu).

**Table 3:** Average thickness of coatings determined by laser diffraction measurement.

	MCC Core [µm]	CaP Core [µm]
Pellet core diameter	287	425
Lf coating thickness	50	95
EC thickness	77	77
Final product diameter	414	597

**Table 4:** The zero-order rate constants [%/week] including 95% confidence intervals (shown in brackets) for different Lf formulations under different storage conditions.

Scheme	Packaging			
**Refrigerator (4 °C)**	**No packaging**	**Cps**	**Cps + Alu**	**Alu**
Spray-dried Lf	0.187 (0.157–0.217)	/	/	/
EC MCC pellets	/	/	0.130 (0.094–0.167)	/
EC CaP pellets	/	/	0.136 (0.099–0.172)	/
**Long-term (25 °C, 60% RH)**	**No packaging**	**Cps**	**Cps + Alu**	**Alu**
Spray-dried Lf	0.685 (0.557–0.812)	/	/	0.162 (0.112–0.212)
EC MCC pellets	0.353 (0.280–0.425)	0.325 (0.240–0.410)	0.206 (0.093–0.318)	0.329 ^a^ (0.247–0.411)
EC CaP pellets	0.327 (0.263–0.391)	0.311 (0.256–0.366)	0.205 (0.145–0.265)	0.193 (0.123–0.263)
**Accelerated (40 °C, 75% RH)**	**No packaging**	**Cps**	**Cps + Alu**	**Alu**
Spray-dried Lf	12.252 (11.717–12.787)	/	0.445 (0.401–0.489)	0.440 (0.405–0.475)
MCC pellets without EC	9.959 (9.703–10.215)	1.207 (1.073–1.340)	0.507 (0.465–0.550)	/
CaP pellets without EC	10.447 (10.228–10.665)	1.228 (1.122–1.339)	0.531 (0.499–0.563)	/
EC MCC pellets	5.393 (5.054–5.733)	2.026 (1.904–2.147)	0.596 (0.554–0.638)	/
EC CaP pellets	4.663 (4.220–5.106)	1.535 (1.410–1.660)	0.611 (0.583–0.640)	/
**Controlled room T (up to 25 °C)**	**Dissolved ^b^**			
Spray-dried Lf	0.600 (0.445–0.754)			
MCC pellets without EC	0.594 (0.420–0.769)			
CaP pellets without EC	0.735 (0.648–0.822)			
EC MCC pellets	1.590 (1.387–1.794)			
EC CaP pellets	1.684 (1.398–1.971)			

Abbreviations: microcrystalline cellulose (MCC), calcium phosphate (CaP), enteric coating (EC), relative humidity (RH), gelatin capsules (Cps), alu-alu packaging (Alu). ^a^ Alu-alu packaging was not sealed correctly and opened during the study; ^b^ Dissolved samples were prepared by dissolving spray-dried Lf or pellets in 50 mM phosphate buffer (pH 6.8) at a concentration of approximately 1 mg/mL; /—not evaluated.

**Table 5:** The moisture content (±SD) in the samples at the beginning and after 26 weeks of stability study determined by Karl Fischer titration.

Conditions	Samples	Packaging	% H_2_O ± SD	
			0 Weeks	26 Weeks
40 °C 75% RH	Spray-dried Lf	No packaging	6.6 ± 0.1	13.7 ± 0.1
		Cps + Alu		8.0 ± 0.2
		Alu		8.4 ± 0.0
	EC MCC pellets	No packaging	4.6 ± 0.1	8.8 ± 0.1
		Cps		9.0 ± 0.1
		Cps + Alu		4.1 ± 0.0
	EC CaP pellets	No packaging	3.8 ± 0.1	6.6 ± 0.1
		Cps		7.7 ± 0.0
		Cps + Alu		5.7 ± 0.1
	MCC pellets without EC	No packaging	4.9 ± 0.1	11.2 ± 0.0
		Cps		11.5 ± 0.3
		Cps + Alu		7.7 ± 0.0
	CaP pellets without EC	No packaging	3.2 ± 0.1	8.4 ± 0.2
		Cps		9.3 ± 0.1
		Cps + Alu		5.6 ± / *
25 °C 60% RH	Spray-dried Lf	No packaging	6.6 ± 0.1	13.4 ± 0.1
		Alu		6.6 ± 0.4
	EC MCC pellets	No packaging	4.6 ± 0.1	9.5 ± 0.0
		Cps		8.6 ± 0.2
		Cps + Alu		4.8 ± 0.0
		Alu		7.6 ± 0.1 ^a^
	EC CaP pellets	No packaging	3.8 ± 0.1	7.1 ± 0.5
		Cps		7.3 ± 0.1
		Cps + Alu		4.0 ± 0.2
		Alu		4.3 ± 0.1
4 °C	Spray-dried Lf	No packaging	6.6 ± 0.1	10.2 ± 0.8
	EC MCC pellets	Cps + Alu	4.6 ± 0.1	4.3 ± 0.1
	EC CaP pellets	Cps + Alu	3.8 ± 0.1	3.8 ± 0.4

* Alu-alu packaging was not sealed correctly and opened during the study.

**Table 6:** Experimentally determined shelf life of the final product.

Storage Conditions	Pellet Core	Shelf Life [Years]	
		Without Confidence Interval	Using 95% Confidence Interval
25 °C, 60% RH	MCC	1.9	1.3
	CaP	1.9	1.6
Refrigerator (4 °C)	MCC	3.0	2.4
	CaP	2.8	2.4

## Data Availability

Data are contained within the article.
